# Assessing the Knowledge, Attitudes and Practices of COVID-19 among Quarantine Hotel Workers in China

**DOI:** 10.3390/healthcare9060772

**Published:** 2021-06-21

**Authors:** Yi-Man Teng, Kun-Shan Wu, Wen-Cheng Wang, Dan Xu

**Affiliations:** 1 College of Modern Management, Yango University, Fuzhou 350015, China; yimanteng@gmail.com (Y.-M.T.); dxu@ygu.edu.cn (D.X.); 2 Department of Business Administration, Tamkang University, Taipei 251301, Taiwan; 3 College of Innovation and Entrepreneurship Education, Yango University, Fuzhou 350015, China; wcwang@go.hwh.edu.tw

**Keywords:** COVID-19, quarantine hotel workers, knowledge, attitudes, practices

## Abstract

During the pandemic, quarantine hotel workers face a higher risk of infection while they host quarantine guests from overseas. This study’s aim is to gain an understanding of the knowledge, attitudes, and practices (KAP) of quarantine hotel workers in China. A total of 170 participants took part in a cross-sectional survey to assess the KAP of quarantine hotel workers in China, during the COVID-19 pandemic. The chi-square test, independent *t*-test, one-way analysis of variance (ANOVA), descriptive analysis, and binary logistic regression were used to examine the sociodemographic factors associated with KAP levels during the COVID-19 pandemic. The results show that 62.41% have good knowledge, 94.7% have a positive attitude towards COVID-19, but only 78.2% have good practices. Most quarantine hotel workers (95.3%) are confident that COVID-19 will be successfully controlled and that China is handling the COVID-19 crisis well (98.8%). Most quarantine hotel workers are also taking personal precautions, such as avoiding crowds (80.6%) and wearing facemasks (97.6%). The results evidence that quarantine hotel workers in China have acquired the necessary knowledge, positive attitudes and proactive practices in response to the COVID-19 pandemic. The results of this study can provide a reference for quarantine hotel workers and their targeted education and intervention.

## 1. Background

The COVID-19 pandemic has made a significant impact on the health and safety of each country’s population, as well as ongoing effects to their economies and societies. The World Health Organization has declared the pandemic a public health emergency of global concern [1]. As positive cases of COVID-19 escalate, hospitals face problems with overcrowding and insufficient isolation space [2]. Australia enforces home-isolation for confirmed cases with mild symptoms and suspected cases; however, this ultimately increases the risk of infection to other household members [3]. To prevent this interaction, they propose that COVID-19 patients with mild symptoms isolate themselves by staying in a hotel. In addition, due to increasing concerns regarding transmitting the virus to their families, healthcare workers [4] need temporary quarantine accommodation.

To mitigate the pandemic, countries around the world have implemented safety measures such as lockdown, social distancing, and mandatory 14-day quarantine periods for citizens and foreign visitors arriving from abroad [5]. The latter resulted in a demand for designated quarantine hotels, as this is where the majority of incoming residents and visitors will stay. As a result, many governments have expropriated hotels to be used as temporary quarantine accommodation: the ‘quarantine hotel.’ Quarantine hotels are a community-based public health intervention designed to mitigate the spread of COVID-19 within the community [6]. The use of quarantine hotels to isolate tourists and returning residents for medical observation over a 14-day period is the hotel industry’s contribution to the control of COVID-19 [7]. Some scholars also argue that quarantine hotels reframe the taken-for-granted business model and goes beyond basic cleaning and hygiene standards to devote greater attention to the protection and safety of the quarantine guests’ physical and psychosocial needs, and better fulfill stakeholder demands [8].

Currently, in China, the COVID-19 epidemic is well-controlled; however, confirmed cases from overseas continue to increase [9]. During the pandemic, while hosting quarantine guests from overseas, quarantine hotel workers face a much higher risk of infection [6] as COVID-19′s main route of transmission is through respiratory droplets and direct contact with confirmed cases. Additionally, the quarantine hotel workload includes following an operation guide, complying with high-standard anti-epidemic and disinfection measures, and implementing quarantine services. The challenge for quarantine hotel workers is not only the increasing workload created by the quarantine hotel operation, but also high psychological stress associated with job insecurity, risk of exposure, and contagion for themselves, their friends, and families.

Public health education was evidenced to be the significant measure to mitigate the spread of the epidemic during the SARS, MERS, and COVID-19 pandemics [10,11]. Previous literature proposes webinars (web-based seminars) as a public health educational tool, and provide a viable method of instruction and education for school personnel who are interested in strategies for improving a school’s wellness environment [12]. Recently, some scholars have also evidenced that webinars offer clear and actionable information to school staff about disease characteristics, adoptable preventive measures, and early detection and control of COVID-19 in primary schools [13]. Public health education may improve the effectiveness of preventive measures in terms of transmission of COVID-19 and other viruses.

To effectively curb the COVID-19 crisis, countries worldwide are continuously promoting different unprecedented preventive measures, including appropriate personal hygiene and public health measures [14]. Incorrect knowledge toward the diseases affects people’s incorrect attitude and practices directly raise the risk of infection. Knowledge, attitudes, and practices (KAP) is a significant educational tool for public health and plays an integral role in determining a society’s readiness to accept behavioral change measures from health authorities [15]. Referring to the articles, people’s KAP towards COVID-19 largely affected adherence to control measures in accordance with KAP theory [16,17,18]. According to the previous studies, assessing the KAP toward COVID-19 would assist in providing better insight to address poor knowledge of COVID-19. This also offers the development of preventive strategies and health promotion programs [11,19]. Previous studies have evidenced the relation of a higher level of knowledge and the practice of preventive measures, as well as the positive relation of attitudes and preventive behaviors [20,21,22]. In the latest articles of KAP regarding COVID-19, they all demonstrated collecting KAP information has long been useful for informing prevention, control, and mitigation measures during the epidemic outbreaks [23].

Prior research provides evidence that the level of KAP possessed by inhabitants dictates the success of the adopted measures [15,18,24,25,26]. Recently, there are some studies investigated KAP towards COVID-19 in different group, such as general residents [19,26,27,28,29,30,31,32,33,34,35,36], healthcare workers [24,37,38,39,40,41], students [9,42,43,44,45,46,47,48], patients [49], hospital visitors [50] and slums [51,52], during the COVID-19 pandemic. Most of KAP studies regarding COVID-19 discuss the group of general residents. The results from these studies found that residents with a high level of knowledge about COVID-19 and positive attitudes toward it tended to have better preventive behaviors and behavioral compliance [19,26]. Furthermore, there are fewer articles that discuss KAP toward the COVID-19 system review and future direction [53,54].

Now, the increased trend in confirmed cases in China indicates they are coming from overseas. There is an urgent need to grasp quarantine hotel staff awareness of COVID-19 at this critical time, as they are providing service to host overseas quarantine guests. To the best of our knowledge, there is no published research concentrated on the KAP of quarantine hotel workers. The literature lacks an examination of quarantine hotel workers’ KAP toward COVID-19 from this perspective and has rarely discussed targeted education and intervention for quarantine hotel workers in order to comply with pandemic control measures. If the quarantine hotel workers’ KAPs are concerned about the virus and factors that affect their attitude and behavior, then this information can inform relevant training and policies during their work and guide them in prioritizing protection and avoiding occupational exposure. To date, peer-reviewed COVID-19 KAP surveys have comprised of a brief online survey among ordinary residents, healthcare workers, adults, students, patients, hospital visitors, and slums, and these surveys are not relevant in hospitality industry settings.

To facilitate the management of the COVID-19 pandemic in the hospitality industry, there is an imperative need to grasp the quarantine hotel workers’ awareness of COVID-19 at this critical time. This study aims to assess quarantine hotel workers’ KAP towards COVID-19 through an online questionnaire survey in China. The implication of this study is to anticipate to guide quarantine hoteliers to develop the key skills in the hotel industries for medical education, as well as anti-epidemic and disinfection standards for their staff during and post-pandemic.

## 2. Materials and Methods

### 2.1. Study Design

This cross-sectional study applied convenience sampling to collect samples from the quarantine hotel employees in Xiamen, Fujian Province, China, during the COVID-19 pandemics, from 20 May to 10 June 2020. There are approximately 50 quarantine hotels in Xiamen, as it is the only city in the Fujian Province with airports receiving international flights. The participating staff came from seven hotels. We called the HR manager of the quarantine hotel and asked them whether they would join the survey. Finally, the HR managers of seven hotels agreed to post the one-page recruitment poster on their WeChat (similar to WhatsApp) employee group chat and invited employees to participate in the survey. The advertisement included a brief introduction, background information, purpose, procedures, declarations of anonymity and confidentiality, and the voluntary nature of taking part. The quarantine hotel workers who understood the content of the survey and agreed to participate in the study were instructed to complete the questionnaire via clicking on the link or scanning the QR code.

### 2.2. Study Instrument

The questionnaire consisted of four sections: (1) demographics—this surveyed participants’ sociodemographic information, including gender, age, education, and monthly income; (2) knowledge about COVID-19; (3) attitude toward COVID-19; and (4) practices relevant to COVID-19.

To measure COVID-19 knowledge, 12 items were adapted from Zhong et al. [26]. There were four items regarding clinical presentations (K1–K4), three regarding transmission routes (K5–K7), and five regarding prevention and control (K8–K12). ‘True,’ ‘false,’ or ‘I don’t know’ responses were offered for these items. Correct answers scored ‘1′ and incorrect/unknown answers scored ‘0.’ The score total range for knowledge items was 0–12, with higher scores indicating better knowledge about COVID-19. Bloom’s cut-off of 80% (≥9.6) was used to determine a better knowledge [55].

Attitudes were measured with a two-item scale developed by Zhong et al. [26]. Participants were asked to state their level of agreement on the successful control of COVID-19 (1 = agree; 0 = No/I don’t know), and confidence in winning the battle against the virus (1 = yes; 0 = No). An ‘I don’t know’ response was considered as a lack of agreement and thus, ‘No’ and ‘I don’t know’ were coupled, as per previous studies [14,40]. In addition, the combinations of responses were considered for each participant. The attitude of the participants who agreed that COVID-19 could be successfully controlled and were confident that China could overcome the pandemic scored ‘1′ and were labeled as ‘optimistic attitude’ [26]. ‘No’ or ‘I don’t know’ responses scored ‘0′ and were labeled ‘negative attitude’ [14].

Practice toward COVID-19 was measured with a two-item scale that was developed by Zhong et al. [26]. Participants were asked to state their current behaviors, e.g., going to a crowded place and/or wearing a mask when going out (yes = 1; No = 0). Participants who agreed they had not been to any crowded places and wore a mask when leaving their home scored ‘1′ and were labeled as ‘good practice’ toward COVID-19. The responses that disagreed scored ‘0′ and were labeled as ‘poor practice’ [14].

### 2.3. Statistical Analysis

The data were organized and analyzed using IBM SPSS Statistics (Statistical Package for the Social Sciences) 22.0 software (IBM, Armonk, NY, USA). The chi-squared test, independent *t*-test, and ANOVA with multiple comparisons between each two categories were done by post hoc analysis. Least significant difference (LSD) was applied to find the differences in KAP between groups for selected demographic variables. To identify related factors, the response binary logistic regression analysis was applied and expressed as odds ratio (OR) and 95% confidence interval (CI), with a significance level of 0.05 (two-tailed). For the final model, the Hosmer–Lemeshow test [56], which measures goodness of fit (*p*-value > 0.05), was considered an appropriate logistic regression model. *p* < 0.05 was considered to indicate significance in all tests. Internal consistency of the questionnaire’s knowledge section revealed Cronbach’s alpha as 0.69, which confirms acceptable internal consistency.

### 2.4. Ethical Consideration

According to the relevant laws and regulations of China and the guidelines of Yango University, an ethics approval was not required for this non-interventional study (e.g., surveys). Nevertheless, after quarantine hotel managers agreed to participate in this study, and ethical approval clearance and informed consent clearance were approved by the Luo, Zhong You, Executive principle of Yango University; hence, an ethical approval was expected. After expressing the principles of Helsinki Declaration, the participants were informed of the purpose of the research and expressed their informed consent. Participants were made clear that their participation is voluntary. The study was harmless to the participants, as no names were used and all data were analyzed anonymously in order to maintain anonymity.

## 3. Results

### 3.1. Respondent Characteristics

In terms of demographics among 170 participants, there were slightly more female respondents in this study (*n* = 99, 58.2%) than there were male (41.8%). In terms of ages, 90 participants were Millennials (53.0%) and 32 participants were Generation Z (18.8%). In total, 103 participants (50.6%) had a junior college and above degree. In terms of departments, 28.8% participants were frontline employees (including front desk and housekeeping departments) and 71.2% participants were logistics support employees (including food & beverage, administration, and security departments). One-hundred twenty-eight participants (75.3%) indicated that their individual monthly income was 6000 RMB or below.

### 3.2. Assessment of COVID-19 Knowledge

Results of the knowledge assessment of quarantine hotel workers regarding clinical presentations, transmission routes, and prevention and control of COVID-19 are shown in Table 1. The mean COVID-19 knowledge score for quarantine hotel workers was 9.78 (Standard deviation: 1.61, range: 0–12). The rate of overall correct answer to COVID-19 knowledge was 81.5% (9.78/12 × 100). The rate of overall correct answer for all quarantine hotel workers ranged between 23.5% and 98.2%. Most workers of the quarantine hotel (98.2%) recognize that human beings who had contact with confirmed cases should be isolated immediately for 14 days. Even so, people are obviously confused about the spread of virus. When asked if eating and contacting wild animals would cause infection, only 23.5% answered correctly (Table 1).

The independent *t*-test and one-way ANOVA analysis were used to assess the differences in knowledge scores among different demographic characteristics. The results showed that there were no significant differences in knowledge scores for all demographic variables (gender, age, education, department, and monthly income) (*p* > 0.05, Table 2).

Initially, most (8/12) knowledge questions about COVID-19 had a high accuracy rate (80% or more) (Table 1). As a result, a cut off knowledge score of ≤9 was set for poor knowledge and ≥10 for good (adequate) knowledge (Table 3). The study found that 62.41% of quarantine hotel workers have good (adequate) knowledge, which implies that a significant proportion of quarantine hotel workers have poor knowledge about COVID-19. Further, binary logistic regression analysis found that female quarantine hotel workers had higher odds of having good (adequate) knowledge at 10% significance level (Table 4).

### 3.3. Assessment of COVID-19 Attitudes

To assess the attitudes toward COVID-19, two questions were used. One asked whether the COVID-19 epidemic would be successfully controlled, which the majority of the quarantine hotel workers agreed with (95.3%). Another asked whether they trusted China to be able to win its battle against the virus, which again, the majority of the quarantine hotel workers agreed with (98.8%). Overall, 94.7% of quarantine hotel workers had an optimistic (positive) attitude toward COVID-19, while 5.3% had a negative attitude (Table 3). In addition, attitudes toward COVID-19 were significantly associated with education level (Table 3). Quarantine hotel workers who had a senior high school/vocational school (vs. middle school and below, OR: 0.020, 95% CI = 0.001–0.682, *p* = 0.030) were more unlikely to have optimistic attitude toward COVID-19 (Table 4).

### 3.4. Assessment of COVID-19 Practices

Quarantine hotel workers were asked two questions in assessment of practices relevant to COVID-19. The first question asked whether or not they agreed that they were avoiding crowded places in recent days; the second was whether or not they agreed that they were wearing face masks when outside the home in recent days. For the first question, 80.6% of quarantine hotel workers reported that they had been avoiding crowded places, whereas the remaining 19.4% had not been. Furthermore, 97.6% of quarantine hotel workers reported wearing a face mask when going out in public in recent days, whereas 2.4% indicated they did not. In addition, 78.2% of quarantine hotel workers had ‘good practice’ relevant to COVID-19. The remaining participants (21.8%) recorded ‘poor practice’ (Table 3). The practice relevant to COVID-19 was not significantly associated with all demographic characteristics (Table 3).

## 4. Discussion

The outbreak of COVID-19 has sent the hotel industry into an unprecedented recession. In this context, the different management policies undertaken by hotel managers are determining the industry’s survival. Quarantine hotel workers’ adherence to control measures is essential, and is largely affected by their KAP towards COVID-19, in accordance with KAP theory. The understanding of quarantine hotel workers’ KAP toward the pandemic is helpful for hoteliers when addressing and implementing effective decision-making frameworks to ensure rapid response to unexpected events that challenge the solvency of their business. Assessing KAP related to COVID-19 provides greater insight, helping to address poor knowledge about the virus and assist with the development of preventive strategies and health promotion programs. KAP studies provide baseline information to determine the type of intervention that may be required to change misconceptions about the virus [14,15,24]. To date, there has been limited published data on quarantine hotel workers’ KAP toward COVID-19. Therefore, it is tremendously important to investigate the KAP of quarantine hotel workers to help guide these efforts.

In China, the overall COVID-19 ‘correct’ knowledge rate among quarantine hotel workers is 81.5%, with an average score of moderate (9.78 ± 1.61). This knowledge score is higher than that of US residents (80%) [34] and the Palestinian population (79%) [57], but lower than the Chinese general population (90%) [26] and Tanzanian residents (84.4%) [11]. This study found that 62.41% of quarantine hotel workers in China have good (adequate) knowledge of COVID-19, which means there is a considerable proportion of quarantine hotel workers who have poor knowledge. Recently, the research results provide evidence that the level of knowledge regarding COVID-19 was proportional to age and years of education [58]. However, this empirical result reveals that there were no significant differences in knowledge toward COVID-19 scores for the demographic variables (age, education, department, and monthly income). That result is not consistent with other studies conducted worldwide, which shows knowledge was significantly differed across age, education level, and income [26,27]. Our study demonstrated the female quarantine hotel workers were more likely to have good (adequate) knowledge compared to men, which is consistent with the contentions of Zhong et al. [26] and Banik et al. [28], and is similar to a cross-cultural KAP study by Ali et al. [59]. This result may be explained by gender difference in related activities, and can also be attributed to the fact that women will experience higher family pressure in their role of caring for families.

As a considerable amount of quarantine hotel workers in China have poor knowledge of COVID-19, quarantine hoteliers should provide extra medical education to identify microbiological characteristics and perform diagnosis, disinfection, and self-protection technology. Recently, some articles have advocated that hoteliers should improve the service of hygiene and cleaning, disinfection, and hygiene activities as the main contents response to the hotel industries development of post-COVID 19 [60,61,62]. Following the scholars, appropriate training in operation guides, complying with anti-epidemic and disinfection standards, and implementing quarantine services is also essential.

This study also found that a large majority of quarantine hotel workers have optimistic attitudes about controlling and overcoming COVID-19 (94.7%). Roughly 95.3% of quarantine hotel workers concurred that COVID-19 will be controlled and 98.8% of respondents believe China can win the battle against the virus. The participants’ high confidence and optimistic attitudes toward the control of COVID-19 will be attributed to the severe measures taken by the government to slow down the epidemic. The staff of quarantine hotels hold a highly optimistic (positive) attitude towards COVID-19, which is consistent with recent studies, in which most participants believe that their country will fight against it, and this epidemic will be treated in Bangladesh [28], Egypt [27], Malaysia [19], and Saudi Arabia [63]. Attitudes toward COVID-19 play efficacy beliefs among the public, significantly impacting on promoting preventive behaviors [64]. This optimism attitude may be the result of strict disease control measures taken by China government, which have strengthened people’s confidence in their approach.

According to the results, most quarantine hotel workers are taking precautions, such as avoiding crowded places (80.6%) and wearing face masks (97.6%) during the COVID-19 pandemic, which indicates a general willingness for quarantine hotel workers to make behavioral changes to prevent COVID-19 infections. This finding is similar to previous KAP studies regarding COVID-19 [65,66], but contradictory to a study conducted among Malaysian people, which found only 51.2% of participants reported wearing a face mask when going out in public [25]. These practices could be because of the restriction on actions imposed by the government that promote the practice of effective measure to combat the spread of COVID-19 [67].

This study focused on grasping the quarantine hotel workers’ KAP towards COVID-19 at this critical time; however, we are aware of some limits of the study. First, this was a rapid survey whereby participants were recruited by the quarantine hotels’ human resources managers posting a one-page advertisement on their staff group chat in WeChat (similar to WhatsApp) inviting workers to participate. We are not able to verify whether this recruitment method was affected by social desirability bias. Second, the participants were only from quarantine hotels in Xiamen city, which may not reflect the actual situation of the workers in Chinese quarantine hotels as a whole. Third, one of the main shortcomings of KAP surveys is that it is difficult to get a standard reference (cut-off point) to classify the study subjects’ knowledge and practice levels. Lastly, due to the lack of infection data in this dataset, it was not possible to document the infection rates among quarantine hotel workers or the patients remaining in their care and, thus, this research could not determine causality between the variables. These problems require further study and resolution.

## 5. Conclusions

At present, the trend in confirmed cases in China indicates that they are coming from overseas. There is an imperative need to grasp quarantine hotel staff awareness of COVID-19 at this critical time, as they are the first to host overseas quarantine guests. The purpose of this study is to explore the characteristics and levels of quarantine hotel workers’ knowledge about COVID-19. To the best of our knowledge, there is a gap in the literature regarding applying the theories of KAP to the hotel industry. This is the first contribution to the assessment of the quarantine hotel workers’ awareness about COVID-19 risks. To enable further progress in this field, a deeper understanding of how KAP theory research in hospitality is required. The findings suggest that most quarantine hotel workers in China have adequate knowledge on COVID-19 and are generally optimistic (positive) in their outlook on overcoming the pandemic.

The survey results provide a general outline of quarantine hotel workers’ COVID-19 prevention practices, which can better prepare quarantine hoteliers when addressing future health crises. The results also highlight future targeted education and intervention for quarantine hotel workers in order to comply with pandemic control measures, such as providing staff with training in anti-epidemic and disinfection standards and implementing quarantine services.

It is anticipated that in the post-pandemic future, the key skills in the hotel industry’s competitive workforce will be medical education and anti-epidemic and disinfection standards. Currently and moving forward, hoteliers will focus their attention on hygiene and cleanliness and promote this message via social media, e.g., ‘We have high standards of cleanliness and hygiene,’ to ensure customers trust the hotel and feel comfortable and safe during their stay.

## Figures and Tables

**Table 1:** Responses to the questionnaire on COVID-19 KAP.

Items	Correct Answer Rate (*n*; %)	Incorrect Answer and ‘I Don’t Know’ Rate (*n*; %)
K1. The main clinical symptoms of COVID-19 are fever, fatigue, dry cough, and myalgia.	159 (93.5)	11 (6.5)
K2. Unlike the common cold, nasal congestion, runny nose, and sneezing are less common among people infected with COVID-19 virus.	113 (66.5)	57 (33.5)
K3. At present, there is no effective treatment in COVID-19, but early symptomatic treatment can help most patients recover from infection.	152 (89.4)	18 (10.6)
K4. Not all persons with COVID-19 will develop into severe cases. Only those who are elderly, have chronic illnesses, and are obese are more likely to be severe cases.	113 (66.5)	57 (33.5)
K5. Eating or contacting wild animals would result in the infection by the COVID-19 virus.	40 (23.5)	130 (76.5)
K6. Persons with COVID-19 cannot pass the virus to others when a fever is not present.	130 (76.5)	23.5 (4.0)
K7. The COVID-19 virus spreads via respiratory droplets from infected individuals.	162 (95.3)	8 (4.7)
K8. Ordinary residents can wear general medical masks to prevent infection from the COVID-19 virus.	162 (95.3)	8 (4.7)
K9. It is not necessary for children and young adults to take measures to prevent infection from the COVID-19 virus.	156 (91.8)	14 (8.2)
K10. To prevent infection by COVID-19, individuals should avoid going to crowded places such as train stations and avoid taking public transportation.	150 (88.2)	20 (11.8)
K11. Isolation and treatment of people who are infected with the COVID-19 virus are effective ways to reduce the spread of the virus.	159 (93.5)	11 (6.5)
K12. People who have contact with someone infected with the COVID-19 virus should be immediately isolated in a proper place. In general, the observation period is 14 days.	167 (98.2)	3 (1.8)
Attitudes	Answer yes rate (*n*; %)	Answer no rate (*n*; %)
A1. Do you agree that COVID-19 will finally be successfully controlled?	162 (95.3)	8 (4.7)
A2. Do you have confidence that China can win the battle against the COVID-19 virus?	168 (98.8)	2 (1.2)
Practice	Answer yes rate (*n*; %)	Answer no rate (*n*; %)
P1. Have you been to any crowded places in recent days?	33 (19.4)	137 (80.6)
P2. Do you wear a mask when you go out in recent days?	166 (97.6)	4 (2.4)

**Table 2:** Relationship between socio-demographic characteristics of the participants and their knowledge scores about COVID-19 (*n* = 170).

Characteristics		Category	Number of Participants (%)	Knowledge Score (Mean ± SD)	*t*/F	*p*-Value
Gender	Male	a	71 (41.8)	9.54 ± 1.52	−1.707	0.090
	Female	b	99 (51.2)	9.96 ± 1.65		
Age	Generation Z	a	32 (18.8)	9.22 ± 2.15	2.529	0.083
	Millennials	b	90 (52.9)	9.88 ± 1.51		
	Generation X	c	48 (28.2)	9.98 ± 1.28		
Education	MSB	a	20 (11.8)	9.75 ± 1.07	0.431	0.731
	SHSVS	b	47 (27.6)	9.85 ± 1.43		
	JC	c	57 (33.5)	9.91 ± 1.84		
	UA	d	46 (27.1)	9.57 ± 1.70		
Department	Frontline	a	49 (28.8)	9.86 ± 1.37	0.385	0.701
	Logistics support	b	121 (71.2)	9.75 ± 1.70		
Income per month RMB ^#^	6000 and below	a	128 (81.5)	9.85 ± 1.53	−0.254	0.800
	6001 and above	b	29 (18.5)	9.93 ± 1.46		

Note: (1) Generation Z = Born 1996+; Millennials = Born 1977–1995; Generation X = Born 1965–1976. (2) MSB = Middle school and below; SHSVS = Senior high school/vocational school; JC = Junior college; UA = Undergraduate and above. (3) ^#^ Exclude ‘I don’t want to talk about it’ participants. (4) *t* = Student’s *t* test, F = analysis of variance (ANOVA) test. (5) Multiple comparisons between each two categories are done by post hoc analysis (Least Significant Difference, LSD).

**Table 3:** Difference in quarantine hotel workers’ KAP toward COVID-19 by demographics (*N* = 170).

Characteristics	Knowledge			Attitude			Practice		
	Poor (*n*; %)	Good (*n*; %)	χ^2^ or *t* (*p*-Value)	Negative (*n*; %)	Optimistic (*n*; %)	χ^2^ or *t* (*p*-Value)	Poor (*n*; %)	Good (*n*; %)	χ^2^ or *t* (*p*-Value)
Overall	64 (37.6)	106 (62.41)		9 (5.3)	161 (94.7)		37 (21.8)	133 (78.2)	
Gender			5.446 (0.020)			0.278 (0.598)			0.043 (0.837)
Male	34 (47.9)	37 (52.1)		3 (4.2)	68 (95.8)		16 (22.5)	55 (77.5)	
Female	30 (30.3)	69 (69.7)		6 (6.1)	93 (93.9)		21 (21.2)	78 (78.5)	
Age			1.555 (0.460)			0.400 (0.819)			2.112 (0.348)
Gen Z	15 (46.9)	17 (53.1)		1 (3.1)	31 (96.9)		10 (31.2)	22 (68.8)	
Millennials	31 (34.4)	59 (65.6)		5 (5.6)	85 (94.4)		18 (20.0)	72 (80.0)	
Gen X	18 (37.5)	30 (62.5)		3 (6.3)	45 (93.8)		9 (18.8)	39 (81.3)	
Education level			1.775 (0.620)			7.045 (0.070)			2.892 (0.409)
Middle school and below	7 (35.0)	13 (65.0)		3 (15.0)	17 (85.0)		4 (20.0)	16 (80.0)	
Senior high school/vocational school	19 (40.4)	28 (59.6)		4 (8.5)	43 (91.5)		8 (17.0)	39 (83.0)	
Junior college	18 (31.6)	39 (68.4)		1 (1.8)	56 (98.2)		11 (19.3)	46 (80.7)	
Undergraduate and above	20 (43.5)	26 (56.5)		1 (2.2)	45 (97.8)		14 (30.4)	32 (69.6)	
Department			0.024 (0.876)			0.094 (0.759)			1.873 (0.171)
Frontline	18 (36.7)	31 (63.3)		3 (6.1)	46 (93.9)		14 (28.6)	35 (71.4)	
Logistics support	46 (38.0)	75 (62.0)		6 (5.0)	115 (95.0)		23 (19.0)	98 (81.0)	
Income per month RMB ^#^			1.300 (0.254)			0.496 (0.481)			1.138 (0.286)
6000 and below	43 (33.6)	85 (66.4)		5 (3.9)	123 (96.1)		24 (18.8)	104 (81.2)	
6001 and above	13 (44.8)	16 (55.2)		2 (6.9)	27 (93.1)		8 (27.6)	21 (72.4)	
Knowledge score	8.25 ± 1.53	10.71 ± 0.68	−14.375 (0.000)	9.33 ± 1.80	9.81 ± 1.60	−0.860 (0.391)	9.78 ± 1.57	9.78 ± 1.63	0.006 (0.995)

Note: (1) Knowledge section total scores range from 0–12, with a cut off level of ≤9 set for poor knowledge and ≥10 for good knowledge. (2) The attitude of the participants who agreed that COVID-19 could be successfully controlled and were confident about China winning against the pandemic scored ‘1’ and was labeled as ‘optimistic attitude’ toward COVID-19. Any other combinations of responses scored ‘0’ and were labeled as ‘negative attitude’ toward COVID-19. (3) The practice of the participants who agreed they had not gone to any crowded places and wore a mask when leaving home in recent days scored ‘1’ and was labeled as ‘good practice’ regarding COVID-19. Any other combinations of responses scored ‘0’ and were labeled as ‘poor practice’ regarding COVID-19. (4) ^#^ Exclude ‘I don’t want to talk about it’ participants.

**Table 4:** Logistic regression analysis for factors associated with good knowledge and optimistic attitude regarding COVID-19 (*N* = 170).

Characteristics	Knowledge		Attitude	
	OR (95% CI)	*p*-Value	OR (95% CI)	*p*-Value
Gender (Reference: Male)				
Female	1.881 (0.922, 3.837)	0.082	0.522 (0.087, 3.139)	0.269
**Age (Reference: Generation Z)**				
Millennials	1.679 (0.605, 4.657)	0.319	9.066 (0.349, 235.311)	0.185
Generation X	1.517 (0.461, 4.989)	0.493	9.656 (0.288, 323.420)	0.206
**Education (Reference: Middle school and below)**				
Senior high school/ vocational school	1.292 (0.355, 4.702)	0.697	0.020 (0.001, 0.682)	0.030 *
Junior college	1.272 (0.469, 3.452)	0.636	0.151 (0.009, 2.462)	0.184
Undergraduate and above	1.964 (0.797, 4.839)	0.142	1.001 (0.058, 17.420)	0.999
**Department (Reference: logistics support department)**				
Frontline	0.723 (0.333, 1.572)	0.413	0.516 (0.079, 3.372)	0.490
**Income per month RMB (Reference: 6000 and below)** ^#^				
6001 and above	0.460 (0.177, 1.197)	0.112	0.062 (0.003, 1.137)	0.061
Hosmer–Lemeshow goodness of fit statistic	8.277	0.309	2.050	0.979

Note: (1) ^#^ Exclude ‘I don’t want to talk about it’ participants; (2) * Statistically significant at *p* < 0.05.

## Data Availability

Data sharing not applicable.
